# Nucleo-cytoplasmic shuttling of 14-3-3 epsilon carrying hnRNP C promotes autophagy

**DOI:** 10.1080/15384047.2023.2246203

**Published:** 2023-08-20

**Authors:** Manlan Guo, Minyi He, Yi Zhang, Weiwen Liu, Min Qi, Zhifeng Liu, Guozhong Yi, Shengze Deng, Yaomin Li, Xuegang Sun, Liang Zhao, Tengxiang Chen, Yawei Liu

**Affiliations:** a Department of Neurosurgery & Medical Research Center, Shunde Hospital, Southern Medical University (The First People’s Hospital of Shunde Foshan), Foshan, China; b Transformation Engineering Research Center of Chronic Disease Diagnosis and Treatment, Department of Physiology, School of Basic Medical Sciences, Guizhou Medical University, Guiyang, Guizhou, China; c Center for Clinical Medical Education, Nanfang Hospital, Southern Medical University, Guangzhou, Guangdong, China; d Department of Oncology, Guizhou Cancer Hospital, Guiyang, Guizhou, China; e Guangdong Provincial Key Laboratory of Molecular Oncologic Pathology, Southern Medical University, Guangzhou, China; f School of Traditional Chinese Medicine, Southern Medical University, Guangzhou, China

**Keywords:** Triptolide, 14-3-3ε, hnRNP C, proliferation, autophagy

## Abstract

Translocation of 14-3-3 protein epsilon (14-3-3ε) was found to be involved in Triptolide (Tp)-induced inhibition of colorectal cancer (CRC) cell proliferation. However, the form of cell death induced by 14-3-3ε translocation and mechanisms underlying this effect remain unclear. This study employed label-free LC-MS/MS to identify 14-3-3ε-associated proteins in CRC cells treated with or without Tp. Our results confirmed that heterogeneous nuclear ribonucleoproteins C1/C2 (hnRNP C) were exported out of the nucleus by 14-3-3ε and degraded by ubiquitination. The nucleo-cytoplasmic shuttling of 14-3-3ε carrying hnRNP C mediated Tp-induced proliferation inhibition, cell cycle arrest and autophagic processes. These findings have broad implications for our understanding of 14-3-3ε function, provide an explanation for the mechanism of nucleo-cytoplasmic shuttling of hnRNP C and provide new insights into the complex regulation of autophagy.

## Introduction

The highly conserved 14-3-3 protein family includes 14-3-3α/β, γ, ε, η, τ/θ, σ, and ζ/δ in mammals.^1^ These proteins are involved in the regulation of various cellular processes, such as metabolism, protein trafficking, signal transduction, apoptosis and cell-cycle regulation.^2–5^ 14-3-3 proteins can interact with many different proteins due to their specific phospho-serine/phospho-threonine binding activity.^6,7^ Our previous studies have revealed that translocation of 14-3-3ε is involved in Tp-induced inhibition of CRC cell proliferation.^8^ Tp, a diterpene triepoxide, is a major active component of extracts derived from the medicinal plant Tripterygium wilfordii Hook F (TWHF), which has been used to treat inflammatory and autoimmune diseases.^9^ An increasing body of evidence from recent experimental studies have shown the potential value of Tp in cancer treatment (breast cancer, colorectal cancer, liver cancer, and pancreatic cancer).^8,10–12^ The pharmacological effects of triptolide involve multiple signal pathways and several cellular targets have been demonstrated, such as Hsp70, nuclear factor-kappa B, vascular endothelial growth factor and RNA polymerase II^13^. Recently, several studies have shown that triptolide mediates leukemia cells and prostate cancer cells death by inducing both apoptosis and autophagy pathways^14,15^. Triptolide has also been reported to suppress HT29 and SW480 colon cancer cells proliferation but did not significantly affect cells apoptosis^16^. However, the form of cell death induced by 14-3-3ε translocation and the mechanisms underlying this effect remains unclear.

**Figure 1.**
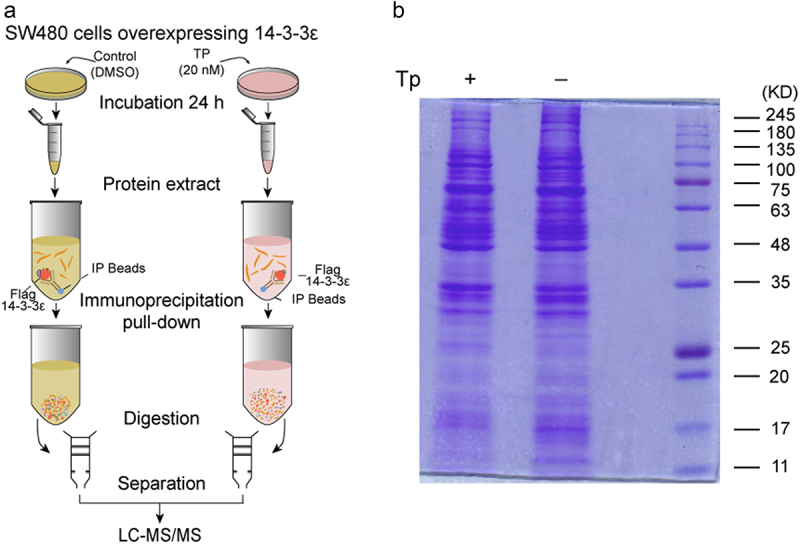
(a) Workflow for identifying 14-3-3ε-interacting proteins in Tp-stimulated SW480 cells. Cells constitutively expressing Flag-14-3-3ε were stimulated by Tp (pink dish), while the same type of cells were left unstimulated (DMSO). The protein complex was purified by FLAG immunoprecipitation (FLAG-IP) with Sepharose® Bead-conjugated FLAG antibody. A label-free method was then used for proteomics analysis. (b) 5% of proteins eluted after IP were evaluated by Coomassie blue staining.

As a member of the least characterized isoform, 14-3-3ε is a highly conserved member of the 14-3-3 family. The major mode of action of 14-3-3ε demonstrated to date is sequestration of 14-3-3-interacting proteins into a compartment, usually the cytoplasm, in which they are unable to carry out their function^6,17^. Thus, in this study, a LC-MS/MS proteomic method was applied to systematically profile the Tp-induced interacting partners of 14-3-3ε in CRC cells (Figure 1a). We provide evidence that the nuclear export of hnRNP C carried out by 14-3-3ε plays an important role in Tp-mediated regulation of CRC cell proliferation, cell cycle and autophagy.

## Materials and methods

### Cell culture, lentiviral infections

SW480 and SW620 cells were purchased from the Chinese Academy of Sciences (Shanghai, China) in December 2015. Two cell lines were authenticated using Short Tandem Repeat analysis in 2016. Cells were maintained in RPMI 1640 medium (Gibco, 8117084, California, USA) supplemented with 10% fetal bovine serum (FBS) and 1% penicillin-streptomycin (Gibco, 1681700, California, USA) in a humidified 37°C incubator with 5% CO_2_. The lentivirals pLent-EF1a-FH-CMV-GFP-P2A-puro and pLent-EF1a-FH-CMV-RFP-P2A-puro were used to introduce genes for over-expression and were purchased from Vigenebio (Shangdong, China). Flag-14-3-3ε was cloned into pLent-EF1a-FH-CMV-GFP-P2A-puro, and wild-type (WT) or mutant (MU) hnRNP C was cloned into pLent-EF1a-FH-CMV-RFP-P2A-puro. To generate cells constitutively expressing Flag-GFP-14-3-3ε, RFP-hnRNP C WT, RFP-hnRNP C MU, Flag-GFP-14-3-3ε + RFP-hnRNP C WT, or Flag-GFP-14-3-3ε + RFP-hnRNP C MU. hnRNP C MU refers to a form of hnRNP C mutated by knocking out a domain (position 160–166, RVSGNTS), which mediates its interaction with 14-3-3ε.

SW480 and SW620 cells were plated into a six-well plate (Corning, NY) at 70–80% confluence and infected by these lentivirals. Two days after infection, stable cell lines were selected with 2.12 mM puromycin (Sigma, P8833, USA) for 3 days and puromycin-resistant cells were subsequently expanded with medium containing 0.53 mM puromycin.

### Reagents

Tp (Nanjing Zelang Medical Technology Co., LTD, Nanjing, China. Accession No. of PubChem Substance: SID: 685302, purity > 98%) was dissolved in Dimethyl Sulphoxide (DMSO) at a stock concentration of 55 M. The dissolved Tp was then added to complete culture medium at final concentration of 20 nM. Cells in complete medium treated with an equal amount of vehicle DMSO served as controls. Chloroquine (CQ, Sigma, C6628, Burlington, Massachusetts, USA) was dissolved in sterile phosphate-buffered saline (PBS, Gibco, 8116481) for a final concentration of 10 μM. 3-methyladenine (3-MA, Sigma, M9281, Burlington, Massachusetts, USA) was dissolved in PBS for a final concentration of 5 mM. MG132 (Selleck, S2619, Houston, Texas, USA) was dissolved in DMSO and diluted to 10 μM before use.

### Immunoprecipitation (IP)

Cells (1 × 10^7^) over-expressing Flag-GFP-14-3-3ε were treated with normal medium or medium containing Tp (20 nM). Twenty-four hours later, the cells were lysed with standard IP lysis buffer (Cell Signaling Technology, Danvers, MA, USA) supplemented with protease inhibitor cocktail (Selleck Chemicals) and Phenylmethanesulfonyl fluoride (PMSF) (Solarbio Inc.,Beijing, China). Protein concentration was determined using BCA assays (Solarbio Inc.,Beijing, China). The cleared lysates were transferred to anti-Flag antibody beads or Protein A+G beads conjugated to IgG antibody (Cell Signaling Technology, #5750, Boston, USA) and incubated at 4°C overnight then washed four times with lysis buffer. For SDS-PAGE and Western blotting, the beads were incubated 3 min with SDS-PAGE loading buffer and boiled for 5 min. For liquid chromatography – tandem mass spectrometry (LC–MS/MS) analysis, the beads were eluted with 0.1 M Glycine (pH 2.6) and neutralized with 1 M Tris (pH 8.5) and collected. One sample of each group was used for LC – MS/MS and replicated for three times. Three samples of each group were used for western blotting verification and replicated for three times. A schematic representation is given in Figure 1.

### Label-free proteomics analysis

SW480 cells constitutively expressing Flag-GFP-14-3-3ε were subjected to a quantitative proteomics approach to screen for dynamic 14-3-3ε interacting partners induced by Tp or not. Briefly, cells constitutively expressing Flag-GFP-14-3-3ε were stimulated by Tp, with cells treated with the vehicle DMSO serving as the control. Following the filter-aided sample preparation (FASP) protocol, immunoprecipitated protein complexes were digested and subjected to LC-MS/MS analysis using a Q Exactive mass spectrometer (ThermoFisher Scientific, Massachusetts, America)^18^.

Two micrograms of each sample were FASP digested. Peptide mixtures were separated by HPLC liquid system EASY-nLC1000 (at a flow rate of 400 nL/min with a 120 min-gradient). Solution A is an aqueous solution of 2% acetonitrile with 0.1% formic acid, and Solution B is an aqueous solution of 84% acetonitrile with 0.1% formic acid. Equilibrated with 100% solution A, the peptide mixtures were first eluted in 45% solvent B for 100 min followed by a linear gradient to 100% solvent B in 108 min, after which solvent B was maintained at 100% for 2 min. MS data were acquired using a data-dependent top 10 method dynamically choosing the most abundant precursor ions from the survey scan (300–1800 m/z) for HCD fragmentation. Determination of the target value is based on predictive Automatic Gain Control (pAGC). Dynamic exclusion duration was 40.0 s. Survey scans were acquired at a resolution of 70,000 at m/z 200 and resolution for HCD spectra was set to 17,500 at m/z 200, and isolation width was 2 m/z. Normalized collision energy was 30 eV and the underfill ratio, which specifies the minimum percentage of the target value likely to be reached at maximum fill time, was defined as 0.1%. This process replicated three times.

MaxQuant（version: 1.3.0.5）was set up to search the human portion of the UniProt database (156639 entries, 2017-01-05) assuming trypsin as the digestion enzyme. The precursor and fragment ion mass tolerances were set as 20 ppm and 0.02 Da, respectively. Two missed cleavages were allowed for trypsin and fixed modification was Carbamidomethylation (Cys). Oxidation (Met) and Acetyl (Protein N-term) were specified as variable modification. The peptides were filtered based on andromeda score and false discovery rate (FDR). The cutoff level for andromeda score was set at 40 (andromeda score > 40). The cutoff level for FDR was set at 0.01 (peptide FDR < 0.01, protein FDR < 0.01). Co-fragmentation was performed according to the algorithm of MaxQuant software^19^. Only proteins meeting these criteria and identified in at least two independent experiments were used. Protein abundance was calculated on the basis of the normalized spectral protein intensity (LFQ intensity).

To compare the between the control and treatment samples, label-free quantification was performed with a minimum of twofold changes to determine the differentially expressed proteins. In addition, the Student’s t-test was employed to identify significant changes between the control and treatment samples among the three biological replicates. *P* value < 0.05 were considered to be significant. The mass spectrometry proteomics data have been deposited to the ProteomeXchange Consortium via the PRIDE^20^ partner repository with the dataset identifier PXD008594.

### Western blotting

Cells were lysed with RIPA buffer, and the protein concentrations were determined by BCA assay (Solarbio, #9c0020, Beijing, China). Total protein (40 μg) was resolved by sodium dodecyl sulfate-polyacrylamide gel electrophoresis on a 12% gel and electrotransferred to polyvinylidene fluoride membranes. The membranes were incubated overnight at 4°C with rabbit monoclonal anti-hnRNP C1+C2 (Abcam, ab133607, Cambridge, England), and anti-LC3A/B, anti-ATG3, anti-ATG5, anti-ATG7, and anti-BECLIN1 (for autophagy detection, Abcam, ab133607, Autophagy Antibody Sampler Kit 4445, England). Alternatively, blots were incubated with rabbit polyclonal anti-14-3-3ε (Abcam, ab43057, Cambridge, England), anti-GAPDH (ABclonal, A10868, Wuhan, China), mouse anti-histone H3 (Beyotime, AF0009, Shanghai, China). Blots were then incubated with the corresponding horseradish peroxidase-conjugated secondary antibodies for 1 h at room temperature. Later, they were probed using the ECL Reagent and visualized by a Western blotting analysis detection system. Relative quantitative measurement of Western blotting was performed using ImageJ software.

### Colocalization assay

Cells constitutively expressing Flag-GFP-14-3-3ε + RFP-hnRNP C WT or Flag-GFP-14-3-3ε + RFP-hnRNP C MU were treated with Tp or vehicle Cells were fixed for 10 min in 4% paraformaldehyde and washed with PBS three times. After removal of PBS, cells were stained with 0.2 mg/mL DAPI. Dishes were stored in the dark at 4°C and observed under confocal laser-scanning microscope (Carl Zeiss, LSM 880 with Airyscan, Oberkochen of Baden-Wurttemberg, Germany).

### Ubiquitination assay

For the in vivo ubiquitination assay, SW480 cells constitutively expressing hnRNP C were transfected with HA-tagged ubiquitin (UB) plasmids and treated with Tp. At 24 h after treatment, cells were treated with 10 μM proteasome inhibitor MG132 for 6 h. Cells were then lysed in cell lysis buffer for Western blotting or IP.

### Cell viability assay

Cells constitutively expressing RFP-hnRNP C WT or RFP-hnRNP C MU were plated into a 96-well plate (Corning, NY) at a density of 2 × 10^3^ cells/well and cultured in serum-supplemented RPMI 1640 medium. After 12 h incubation, the cells were treated with Tp (20 nM) or DMSO. Cells in each well were incubated with 10 µl Cell Counting Kit-8 (CCK8, Dojindo, Kumamoto, Japan) diluted in a serum-free medium for another 1 h at 0, 12, 24, 48, and 72 h post-Tp treatment. The optical density (OD) values were detected at 450 nm with a microplate reader (Molecular Devices, MAX190, Silicon Valley, California, USA). CCK-8 data are expressed as the means ± SD, and the experiments were repeated in triplicate.

### Cell-cycle analysis

Cells constitutively expressing RFP-hnRNP C WT or RFP-hnRNP C MU were treated Tp for 24 h. The cells were washed twice with PBS, and the cell concentration was adjusted to 1 × 10^6^/ml. One-milliliter cell suspensions were fixed with 70% ice-cold ethanol at 4°C overnight. After washing again twice in PBS, cells were treated with 100 μl RNase A in a 37°C water bath for 30 min. Then, 400 μl propidium iodide (Cell cycle assay kit, KGA511, Jiangsu, China) was added and incubated for 30 min at room temperature. The distribution of cell-cycle stages was measured by flow cytometry.

### LC3B assay and transmission electron microscope (TEM) imaging for autophagy detection

Briefly, the plasmid pEGFP-C2-LC3B was constructed and transfected using Lipofectamine 2000 (Invitrogen, 1803713) into cells constitutively expressing RFP-hnRNP C WT or RFP-hnRNP C MU. Two days after transfection, the transfected cells were treated with Tp for 24 h and/or autophagy inhibitor then observed by confocal laser-scanning microscope.

Cells constitutively expressing RFP-hnRNP C WT or RFP-hnRNP C MU were treated with Tp for 24 h were fixed with 2.5% glutaraldehyde, washed, and postfixed in osmium. Following dehydration by serial dilutions of ethanol, cells were stained with uranyl acetate and embedded in culture dishes. Thin sections of the cured blocks were cut with a diamond knife, stained with lead citrate, then observed and photographed by TEM (HITACHIH-7650, Tokyo, Japan).

### Statistical analysis

Data were analyzed using SPSS version 23.0 software (SPSS, Chicago, USA). Analysis of Student’s t-test or one-way analysis of variance (ANOVA) was used to determine the statistical significance of differences. All experiments were carried out in triplicate. A value of *P* < .05 was considered statistically significant.

## Results

### Proteomic screening for Tp-induced 14-3-3ε interacting partners

Five percent of proteins eluted after IP were evaluated by Coomassie blue staining (Figure 1b), and the rest were analyzed by label-free proteomics. In MS analysis, protein quantitative analyses generally use the shotgun method to collect original data. The shotgun method is an unlabeled quantitative method based on MS1 data integration to calculate the integral of each peptide signal on the LC/MS chromatogram. Qualitative analysis of proteins were performed using the NCBInr and UniProt databases. LC-MS/MS proteomics method was conducted to investigate the Tp-induced interacting partners of 14-3-3ε in SW480 cells. We treated Flag-GFP-14-3-3ε-SW480 cells with Tp or DMSO for 24 h and harvested total protein for proteomic analysis. As a result, A total of 168 significantly differential proteins were identified in Tp-stimulated Flag-GFP-14-3-3ε-SW480 cells (Table S1), with 84 proteins upregulated and 84 proteins downregulated compared with the control group.

### Verification of 14-3-3ε interaction with hnRNP C

**Figure 2.**
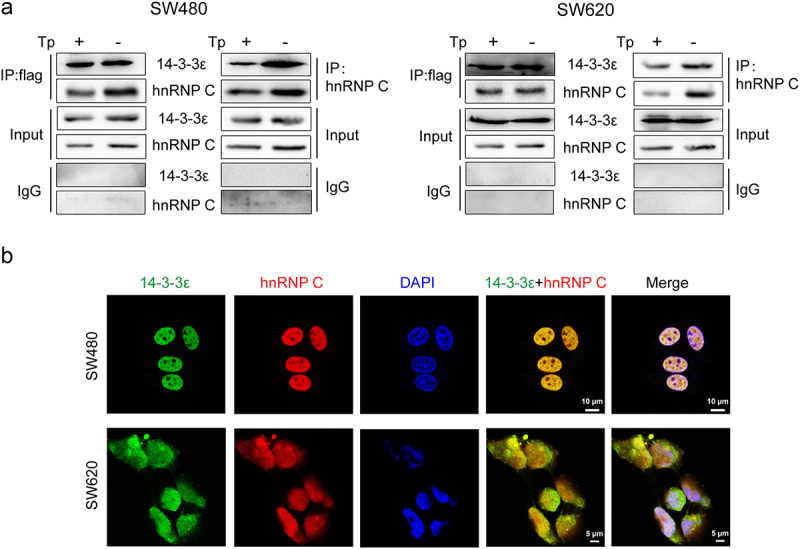
Validation of the interaction of 14-3-3ε and hnRNP C in SW480 and SW620 cells. (a) co-IP. Cells constitutively expressing Flag-14-3-3ε were stimulated with or without Tp (20 nM) for 24 h. The cell lysates were immunoprecipitated with anti-Flag beads and the immunoprecipitates were analyzed by immunoblotting against anti-Flag, anti-14-3-3ε or anti-hnRNP C. Conversely, the cell lysates were immunoprecipitated with anti-hnRNP C antibody-coated beads and the immunoprecipitates were analyzed by immunoblotting against anti-hnRNP C and 14-3-3ε. Inputs represent 5% of the total lysates. IgG beads were used as a control. Blots are representative of at least three independent experiment, data from which was pooled for densitometric analysis. (b) co-localization of 14-3-3ε and hnRNP C in SW480 and SW620 cells was viewed by laser confocal microscopy.

Among the partners we identified, hnRNP C protein had also been shown to be downregulated at the protein level in Tp-induced SW480 cells in our previous study^8^. Song et al. also found that HNRNPC protein levels were downregulated after Tp (40 nM) treatment of HCT116 cells for 48 h^21^. Moreover, hnRNP C possesses phosphopeptides with sequences (160–166, RVSGNTS) similar to the 14-3-3 binding motifs RX1-2SX2-3pS^22,23^. Thus, the interaction between hnRNP C and 14-3-3ε was verified by co-IP and confocal microscopy in Tp-treated SW480 and SW620 cells. The total protein contents of cells constitutively expressing Flag-GFP-14-3-3ε treated with Tp or DMSO were extracted and subjected to IP with anti-Flag antibody-conjugated Sepharose beads then probed by Western blotting. The results showed that hnRNP C co-precipitated with 14-3-3ε (Figure 2a). Fluorescence experiments showed that GFP-14-3-3ε and RFP-hnRNP C co-localize in the nucleus (Figure 2b).

### hnRNP C protein is exported from the nucleus by 14-3-3ε and is degraded by ubiquitination in the cytoplasm

**Figure 3.**
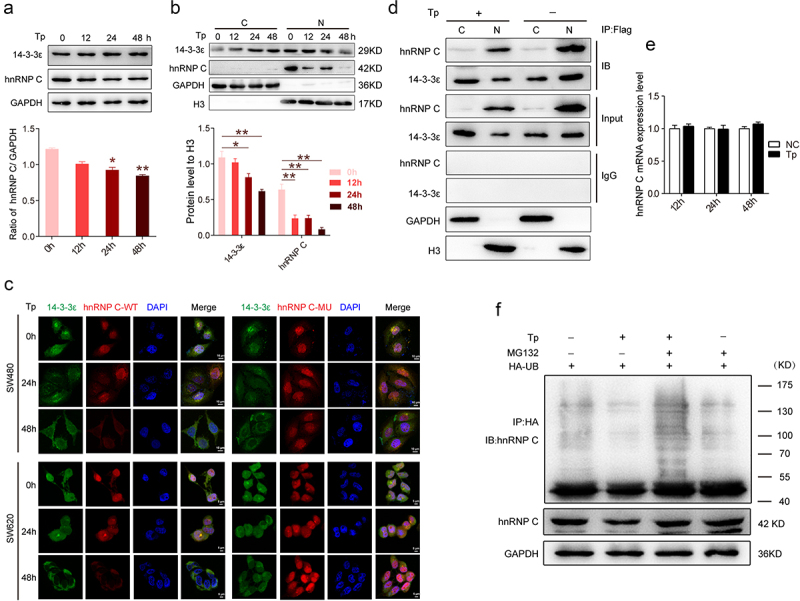
Nucleo-cytoplasmic shuttling of hnRNP C was carried by 14-3-3ε and degradation by ubiquitination. (a) SW480 cells were treated with 20 nM Tp for 12, 24 or 48 h, or with the vehicle DMSO as a control. (b) Nuclear (N) and cytoplasmic (C) protein fractions were isolated. Western blotting analysis of hnRNP C in the indicated cells in response to Tp treatment. GAPDH and histone H3 served as the internal loading controls. The lower histogram shows densitometric analysis of Western blotting results. Results are shown as means (*n* = 3 biologically independent samples); error bars represent the mean ± standard deviation (SD) as indicated. Analysis of one-way analysis of variance (ANOVA) was used to determine the statistical significance of differences (*, *P* < .05; **, *P* < .01). (c) Immunofluorescence assays were used to show the subcellular localization of hnRNP C and 14-3-3ε and semi-quantification of hnRNP C in SW480 cells constitutively expressing hnRNP C-WT or hnRNP C-MU treated with 20 nM Tp for 0, 24 and 48 h. (d) SW480 cells were treated with 20 nM Tp for 24 h, or with the vehicle DMSO as a control. Nuclear (N) and cytoplasmic (c) protein fractions were isolated. Nuclear (N) and cytoplasmic (c) proteins were immunoprecipitated with anti-Flag beads and the immunoprecipitates were analyzed by immunoblotting against anti-14-3-3ε or anti-hnRNP C. Inputs represent 5% of the total lysates. IgG beads were used as a control. (e) SW480 cells were treated with 20 nM Tp for 12, 24 or 48 h, or with the vehicle DMSO as a control. The mRNA levels of hnRNP C were detected by q-PCR. (f) SW480 cells were treated with 20 nM Tp for 48 h, or with the vehicle DMSO as a control. Cells were then transfected with HA-UB and treated with MG132 for 6 h before harvesting. The ubiquitinated hnRNP C proteins were pulled down with anti-hnRNP C antibody and immunoblotted with anti-UB antibody. GAPDH served as the internal loading control.

Previous studies indicated that MALAT‐1 interacted with hnRNP C and facilitated its cytoplasmic translocation in the G2/M phase, thereby regulating the progress of the cell cycle^24^. We decided to examine if hnRNP C protein is exported from the nucleus by 14-3-3ε. SW480 cells were treated with Tp (20 nM) for 0, 12, 24, 48 h, then nuclear (N) and cytoplasmic (C) protein fractions were extracted separately. Total, nuclear, and cytoplasmic protein contents of 14-3-3ε and hnRNP C were detected by Western blotting. Our results showed that nuclear protein level of both 14-3-3ε and hnRNP C decreased following Tp treatment and a small amount hnRNP C protein could be detected following its shuttling out of the nucleus (Figure 3b). This is in sharp contrast to the distribution of H3 and GAPDH, which are known localized predominately in the nucleus or the cytoplasm. Moreover, the total protein level of hnRNP C decreased following Tp treatment (Figure 3a). Next, IP experiments performed using nuclear or cytoplasmic protein fractions also detected more 14-3-3ε-hnRNP C complexes in the nuclear protein fractions after DMSO treatment, whereas fewer complexes were detected in nuclear protein fractions after Tp treatment. We also found that few complexes in cytoplasmic protein fractions after Tp treatment. To strengthen our findings, SW480 and SW620 cells constitutively expressing RFP-hnRNP C WT or RFP-hnRNP C MU were assayed by the fluorescence experiment (Figure 3c). The results also demonstrated that both 14-3-3ε and hnRNP C were translocated into cytoplasm, and hnRNP C protein expression was downregulated following Tp treatment in cells harboring hnRNP C (WT), but not observed in cells harboring hnRNP C (MU) (Figure 3d).

Further, both Western blotting and fluorescence experiments showed that the levels of hnRNP C in total and nuclear protein fractions decreased with Tp treatment (Figure 3a-d). However, quantitative PCR analysis revealed that Tp did not affect hnRNP C mRNA levels (Figure 3e). These results suggest that hnRNP C may be degraded in the cytoplasm following Tp treatment. Ubiquitination is an essential protein modification that influences eukaryotic processes by promoting substrate degradation. Because the hnRNP C sequence contains a ubiquitination modification site, lysine residue K205, we hypothesized that hnRNP C was being ubiquitinated and degraded. To confirm this probability, Ubiquitinated hnRNP C could be detected in SW480 cells overexpressing HA-ubiquitin and increased ubiquitination was observed with the proteasome inhibitor MG132 in the presence of Tp. These results indicated that hnRNP C was degraded in the cytoplasm of SW480 cells after Tp treatment via a proteasome-dependent pathway (Figure 3f). Taken together, these data demonstrated that hnRNP C protein is exported from the nucleus by 14-3-3ε and was ubiquitinated and degraded by proteasomes in the cytoplasm.

### hnRNP C protein translocation is essential for Tp-induced proliferation inhibition and cell cycle arrest

**Figure 4.**
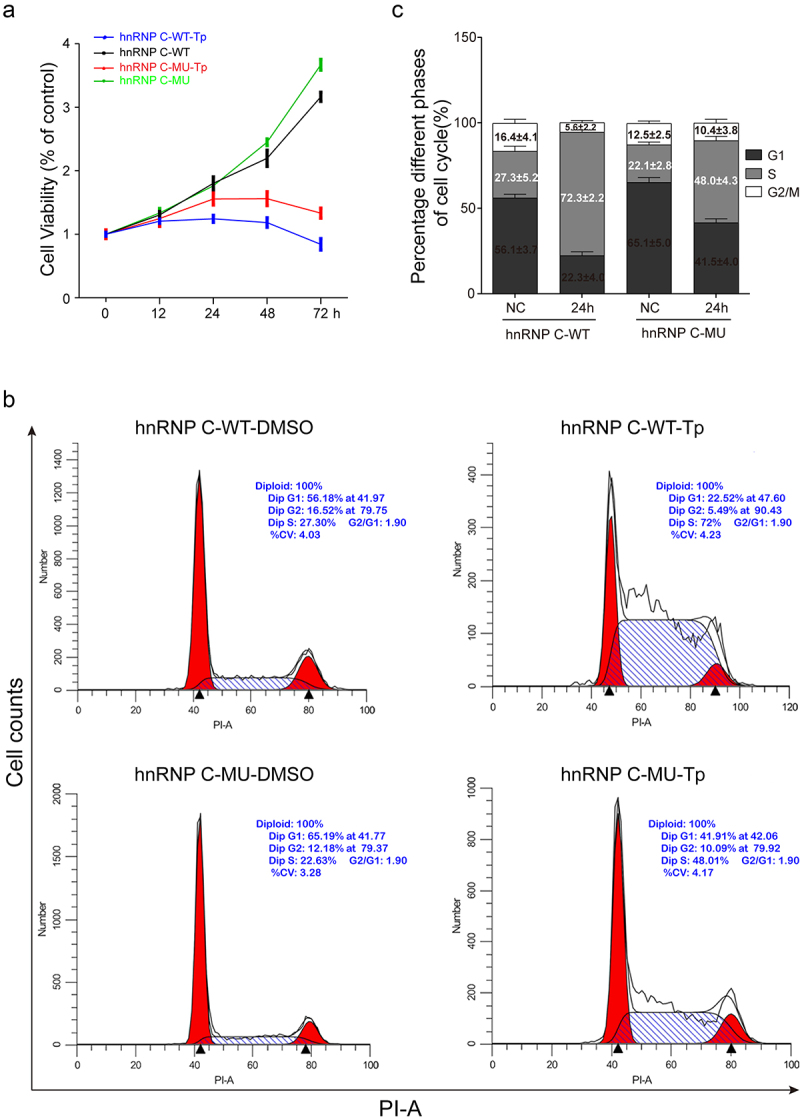
The impact of nucleo-cytoplasmic shuttling of hnRNP C on cell proliferation and the cell cycle. (a) following Tp treatment, the viability of SW480 cells harboring hnRNP C -WT or -MU was evaluated by CCK-8 assay. (b) following 24 h Tp treatment, the cell cycle of SW480 cell harboring hnRNP C -WT or -MU was evaluated by propidium iodide (PI) staining and flow cytometry. (c) cell cycle analysis after Tp treatment. The data are expressed as the mean ± standard deviation. From at least three independent experiments.

We previously demonstrated that translocation of 14-3-3ε is involved in Tp-induced inhibition of CRC cell proliferation. Intriguingly, we observed that hnRNP C was exported out of the nucleus by 14-3-3ε and degraded by ubiquitination. To further examine the effect of hnRNP C shuttles from the nucleus to the cytoplasm on cell proliferation and the cell cycle when cells are treated with Tp. SW480 cells constitutively expressing RFP-hnRNP C wild-type or RFP-hnRNP C mutant-type were assayed by the CCK-8 assay and flow cytometry. Our results revealed that the proliferation capability of cells harboring hnRNP C (MU) was greater than that of cells harboring hnRNP C (WT) following 24 h Tp treatment (Figure 4a). Flow cytometry cell cycle assay showed that Tp arrests SW480 cells in the S phase. Compared with cells harboring hnRNP C (MU), the percentage of cells harboring hnRNP C (WT) in the S phase increased following treatment (Figure 4b-c).

### hnRNP C Protein Translocation is Essential for Tp-induced Autophagy

**Figure 5.**
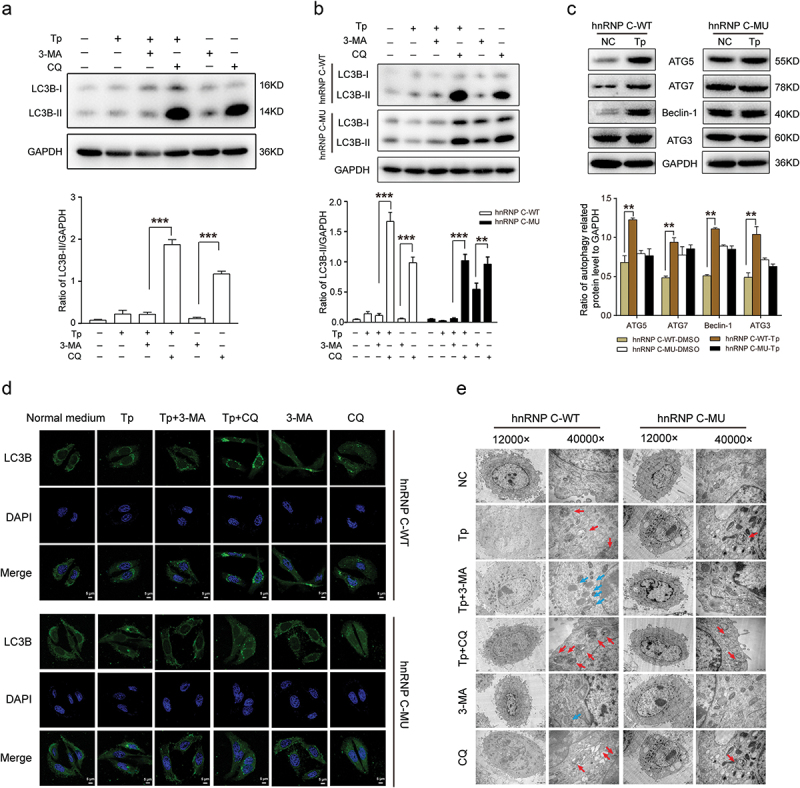
The impact of nucleo-cytoplasmic shuttling of hnRNP C on autophagy. (a) Conversion of LC3B-I to LC3B-II in SW480 cells treated with Tp was detected by Western blotting. GAPDH served as the internal loading control. The histogram shows densitometric analysis of Western blotting results. Results are shown as means (*n* = 3 biologically independent samples); error bars represent the mean ± standard deviation (SD) as indicated. Analysis of one-way analysis of variance (ANOVA) was used to determine the statistical significance of differences (**, *P* < .01; *** *P* < .001). (b) Conversion of LC3B-I to LC3B-II and （c）expression of autophagy-related proteins in SW480 cells harboring WT- and MU-hnRNP C were detected by Western blotting. GAPDH served as the internal loading control. The histogram shows densitometric analysis of Western blotting results. Results are shown as means (*n* = 3 biologically independent samples); error bars represent the mean ± standard deviation (SD) as indicated. Analysis of one-way analysis of variance (ANOVA) was used to determine the statistical significance of differences (** *P* < .01，*** *P* < .001). (d) SW480 cells constitutively expressing hnRNP C-WT or hnRNP C-MU were transfected with GFP-LC3B and treated with 20 nM Tp for 24 h. GFP-LC3B was detected by laser confocal microscopy to measure autophagy levels. (e) representative transmission electron microscope (TEM) images of SW480 cells constitutively expressing hnRNP C-WT or hnRNP C-MU treated with 20 nM Tp for 24 h. Blue arrows indicate formation of autophagic vacuoles. Red arrows indicate formation of lysosomes.

To determine whether autophagy plays a role in Tp-treated cells, we treated the autophagic activity in the SW480 cells with Tp for 24 h. As shown in Figure 5a, the accumulation of LC3B-II induced by Tp was decreased by 3-MA (3-Methyladenine is a selective autophagy inhibitor), but increased by CQ (Chloroquine is a classic inhibitor of autophagy that blocks the binding of autophagosomes to lysosomes). Next, to further validate that the change in autophagy following the nucleo-cytoplasmic shuttling of 14-3-3ε and hnRNP C, SW480 cells constitutively expressing RFP-hnRNP C WT or RFP-hnRNP C MU were assayed by western blotting, LC3B fluorescence detection and Transmission electron microscope (TEM). Western blotting analysis in cells harboring hnRNP C (WT) showed that the accumulation of LC3B-II induced by Tp was decreased by 3-MA, but increased by CQ. However, Tp did not induce any significant changes in LC3B-II accumulation in cells harboring hnRNP C (MU) (Figure 5b). Then, compared with cells harboring hnRNP C (MU), we found that key autophagy-related proteins, such as ATG3, ATG5, ATG7, and Beclin-1 were significantly upregulated in cells harboring hnRNP C (WT) (Figure 5c). Immunofluorescence assays were used to examine the autophagic induction of Tp in cells harboring hnRNP C (WT) or hnRNP C (MU). As shown in Figure 5a, significant fluorescence signals of the mostly autophagy-specific protein LC3 were observed in the Tp-treated cells with puncta accumulation. The result indicated that the punctate staining of LC3B significantly increased after 24 h of treatment with Tp (Figure 5d), and this effect was suppressed by 3-MA. However, in SW480 cells harboring hnRNP C (MU), Tp had no significant autophagy-inducing effects within 24 h. TEM further supported the induction of autophagy by Tp in cells harboring hnRNP C (WT), but not in cells harboring hnRNP C (MU) (Figure 5e). Taken together, these data demonstrated that Tp induced CRC cell autophagy and that over-expression of hnRNP C (MU) reversed autophagy induction.

## Discussion

The goal of the present study is to understand the role of 14-3-3ε in Tp-induced inhibition of CRC cell proliferation. Our results have shown that 14-3-3ε is a regulation factor of autophagy and have provided mechanistic details regarding the nuclear export of hnRNP C carried by 14-3-3ε.

14-3-3 proteins are abundant, powerful and universal regulators of various cellular processes. Their regulatory functions are based on specific recognition of phosphorylated motifs in their partners. Due to the specific recognition of the phosphorylation state of its partners, 14-3-3 proteins bind their targets to stabilize their structure, control their phosphorylation and degradation, and affect their localization and distribution between different cell compartments, thus controlling their interactions with other protein partners. 14-3-3 proteins possess a wide interactome, and they are involved in the regulation of many vital processes such as signal transduction, cell cycle control, apoptosis, transcriptional regulation, cytoskeleton rearrangements, protein localization, trafficking and degradation, exocytosis and endocytosis, and many others^2,3,25,26.^

The major mode of action of 14-3-3ε demonstrated so far is the sequestration of 14-3-3-interacting proteins into a compartment, usually the cytoplasm, in which they are unable to carry out their functions. Based on this mode of action, we sought to identify 14-3-3ε partners in Tp-treated CRC cells using an LC-MS/MS approach. Among the partners we identified, hnRNP C protein had also been shown to be downregulated at the protein level in Tp-induced SW480 cells in our previous study^8^. Moreover, hnRNP C possesses phosphopeptides with sequences (160–166, RVSGNTS) similar to the 14-3-3 binding motifs RX_1–2_SX_2–3_pS^22,23^. Thus, we deduced that the interaction between 14-3-3ε and hnRNP C may play an important role in CRC proliferation inhibition, and we validated the interaction of 14-3-3ε with hnRNP C.

hnRNPs represent a large family of RNA-binding proteins (RBPs) that contribute to multiple aspects of nucleic acid metabolism, including alternative splicing, mRNA stabilization, and transcriptional and translational regulation.^25–28^ There are over 20 types of hnRNPs, the functions of which vary according to their cellular localization.^29,30^ The mechanisms that regulate their nucleo-cytoplasmic shuttling are, therefore, of extreme importance. Most hnRNP proteins possess a conventional nuclear localization signal (NLS) and are predominantly present in the nucleus during the steady state.^31–34^ Nuclear export of the shuttling hnRNP proteins is mediated by nuclear export signals (NESs). Former studies regarded hnRNP C proteins as representative of the non-shuttling group of hnRNP proteins. hnRNP C proteins are restricted to the nucleus not only because they lack an NES but also because they bear a nuclear retention sequence (NRS) that can override NESs.^35^ Later studies revealed the relationship between nuclear translocation of hnRNP C and cell cycle regulation^36^. For example, Kim et al. reported that hnRNP C translocates from the nucleus to the cytoplasm during the G2/M phase to increase the IRES-dependent translation of c-Myc mRNA.^37,38^ Furthermore, another study also reported that MALAT-1 interacts with hnRNP C. Downregulation of MALAT-1 expression compromised the nuclear translocation of hnRNP C in the G2/M phase and resulted in G2/M arrest.^24^ However, the mechanism of nucleo-cytoplasmic shuttling has remained unclear. Our results show that 14-3-3ε undertakes the nucleo-cytoplasmic shuttling of hnRNP C.

Interestingly, in this study, qPCR analysis revealed that Tp did not affect hnRNP C mRNA levels, yet the protein level of hnRNP C decreased, and a small amount hnRNP C protein could be detected following its shuttling out of the nucleus. These results suggested that hnRNP C may be degraded in the cytoplasm. The ubiquitin-proteasome pathway (UPP) is the major non-lysosomal proteolytic pathway within cells^39^, and we theorized that ubiquitination may be responsible for the reduction of hnRNP C protein. Bioinformatics analysis indicates that there is a ubiquitination modification site, lysine residue K205, in the hnRNP C sequence (https://www.uniprot.org/uniprotkb/P07910/entry). Matunis et al. found that hnRNP C proteins are modified by small ubiquitin-related modifiers (SUMOs) at K237. However, they proposed that SUMOylation of hnRNP C occurs at the nuclear pore complexes (NPC, either at the cytoplasmic filaments or at the nucleoplasmic basket) and decreases their binding to nucleic acids but does not cause them to be exported into the cytoplasm or degraded^40^. The present study demonstrates that hnRNP C protein is ubiquitinated and degraded by proteasomes in the cytoplasm. This result explains why hnRNP C cannot shuttle back to the nucleus and loses its function.

The effect of nucleo-cytoplasmic shuttling of 14-3-3ε carrying hnRNP C on CRC cells was then investigated. We found that the export of hnRNP C carried by 14-3-3ε mediated proliferation inhibition, part cell cycle arrest at the S phase, and autophagy. It is necessary to note that some studies indicated that Tp may induce apoptosis^41,42^, but Liu et al. have found that Tp inhibited SW480 cells proliferation independent of induction of cells apoptosis. Our results show instead a significant autophagy response in Tp-induced SW480 cells. Little is known about the exact roles of 14-3-3ε and hnRNP C in autophagy. Sai et al. reported that autophagic activity was promoted by 14-3-3ε siRNA transfection, but the mechanism was not further discussed^43^. Zhong et al. showed that abolishing 14-3-3ε with CLIC4 interaction resulted in Beclin 1 overactivation, which further activated autophagy^44^. In this work, we provide the evidence that the nucleo-cytoplasmic shuttling of 14-3-3ε-hnRNP C releases the inhibition of hnRNP C on autophagy. This result offers a new insight into autophagy regulation.

## Supplementary Material

Supplemental Material
