# Supplementary material for: Nucleo-cytoplasmic shuttling of 14-3-3 epsilon carrying hnRNP C promotes autophagy
Source: Cancer Biol Ther. 2023 Aug 20;24(1):2246203. doi: 10.1080/15384047.2023.2246203 (PMC10443976; doi:10.1080/15384047.2023.2246203)
Supplement: Supplemental Material [file KCBT_A_2246203_SM0074.docx]

## ***Supplementary Materials***


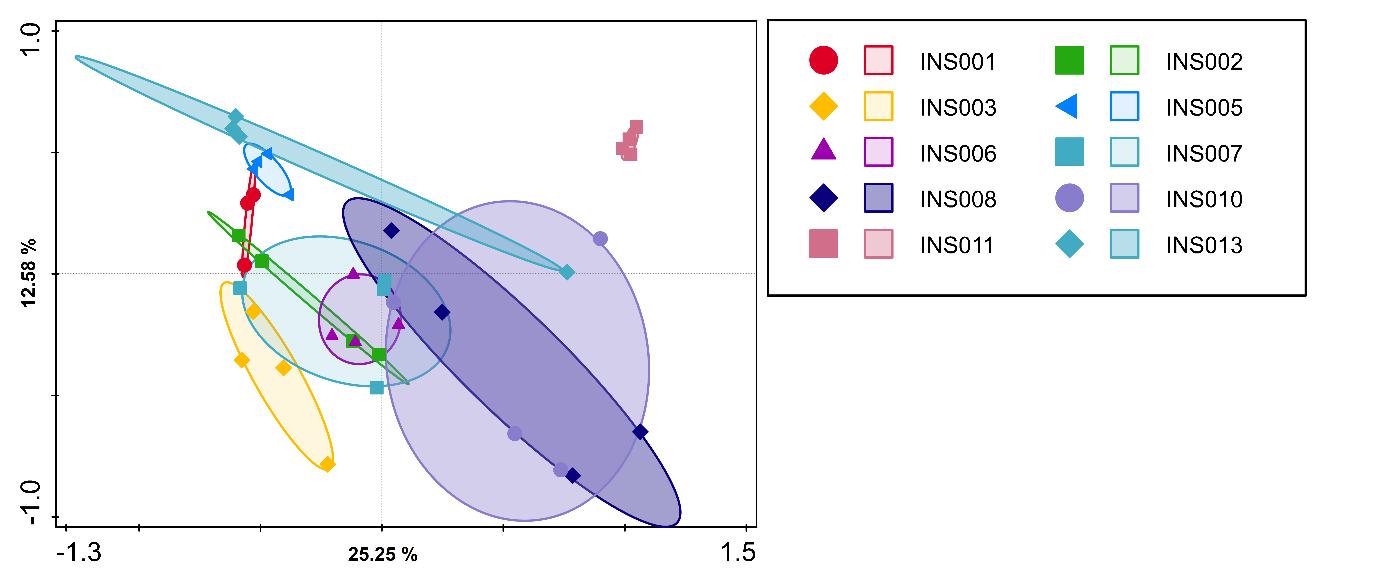


Supplementary figure 1: redundancy analysis of the overall metataxonomic data distribution of the ileostomy effluents microbiota. The microbial composition of the ileostomy samples was evaluated via 16S rDNA sequencing. Subject ID explains 49.92% of the total data variation at the species level (p-value=0.002), illustrating the individuality of the small intestinal microbial community. In addition, the samples obtained post-consumption of the L. rhamnosus fermented dairy product could not be significantly distinguished from the baseline sample (taken prior to consumption).


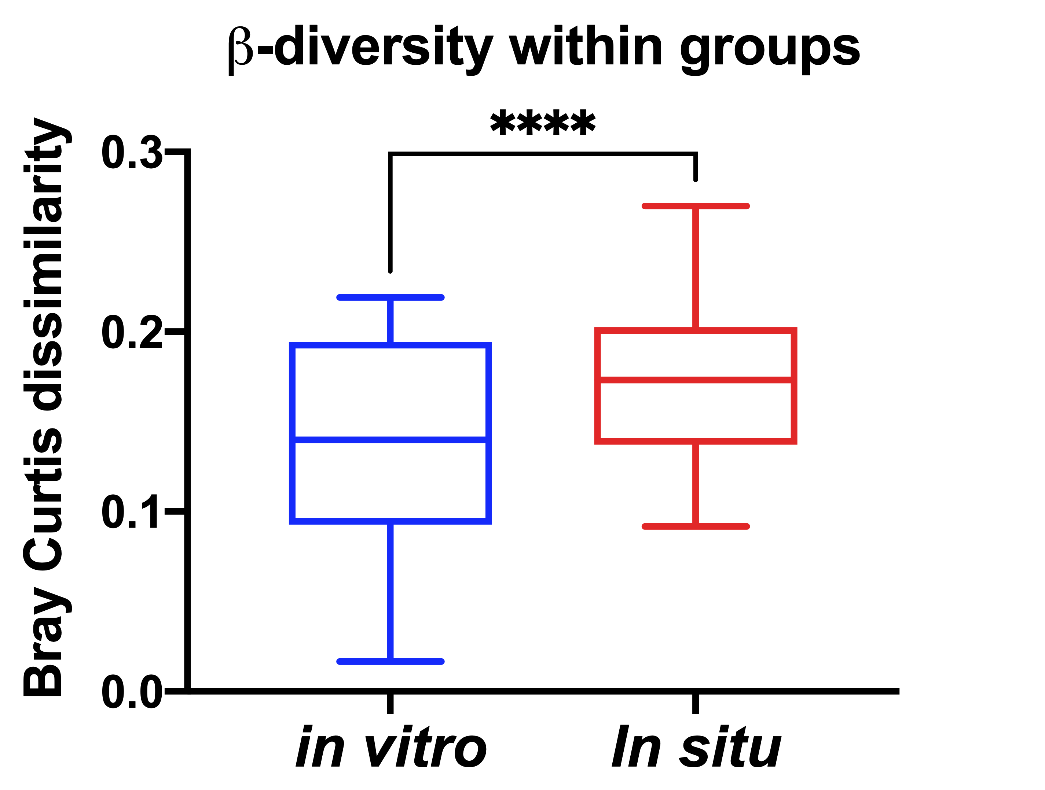


*Supplementary figure 2:* *β-diversity of the* L. rhamnosus *transcriptome In vitro and In situ. Bray-Curtis dissimilarity has been calculated within the same group and the two groups compared resulting in a significant difference between the In vitro and In situ groups (**Mann-Whitney test, p<0.0001). The In situ samples showed higher dissimilarities among them, reflecting the individuality of the niche and ecosystem of the small intestinal tract.*


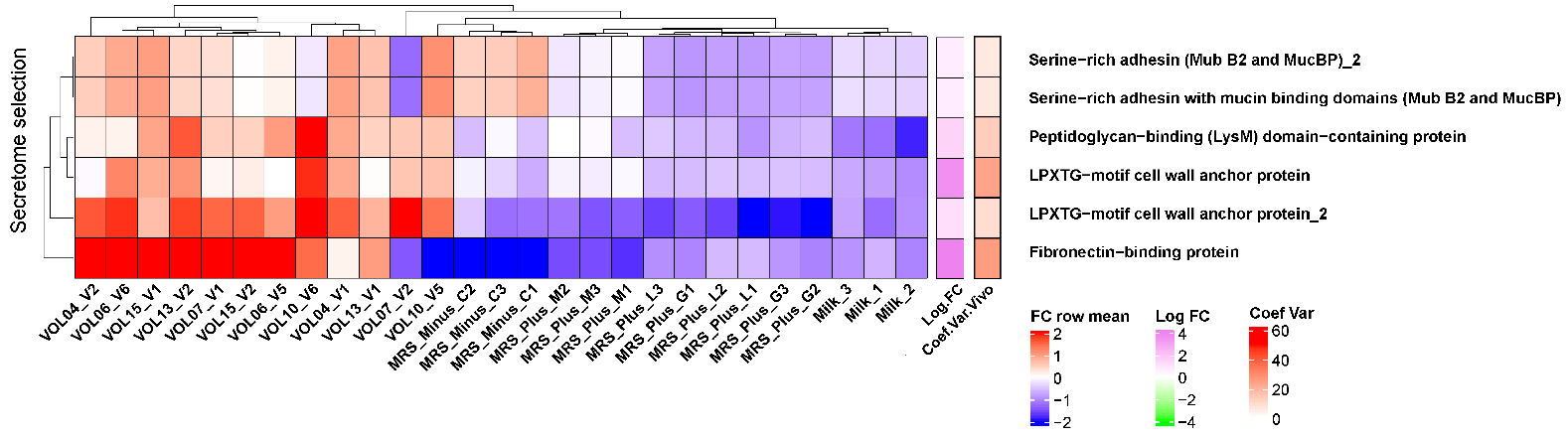


Supplementary figure 3: heatmaps of selected Secretome gene expression. Genes of particular interest were selected on basis of functional domains that are annotated to be involved in bacterial adhesion such as a fibronectin-binding domain, peptidoglycan-binding (LysM) domain, serine-rich adhesins-encoding genes containing mucin binding domains as well as a bacterial lectin, and/or LPXTG-motif cell wall anchored domain. EdgeR, FDR adjusted p-value: 0.05 in situ vs in vitro conditions. The colours represent fold change from the row mean, hierarchical clustering: complete linkage based on Euclidian distances.


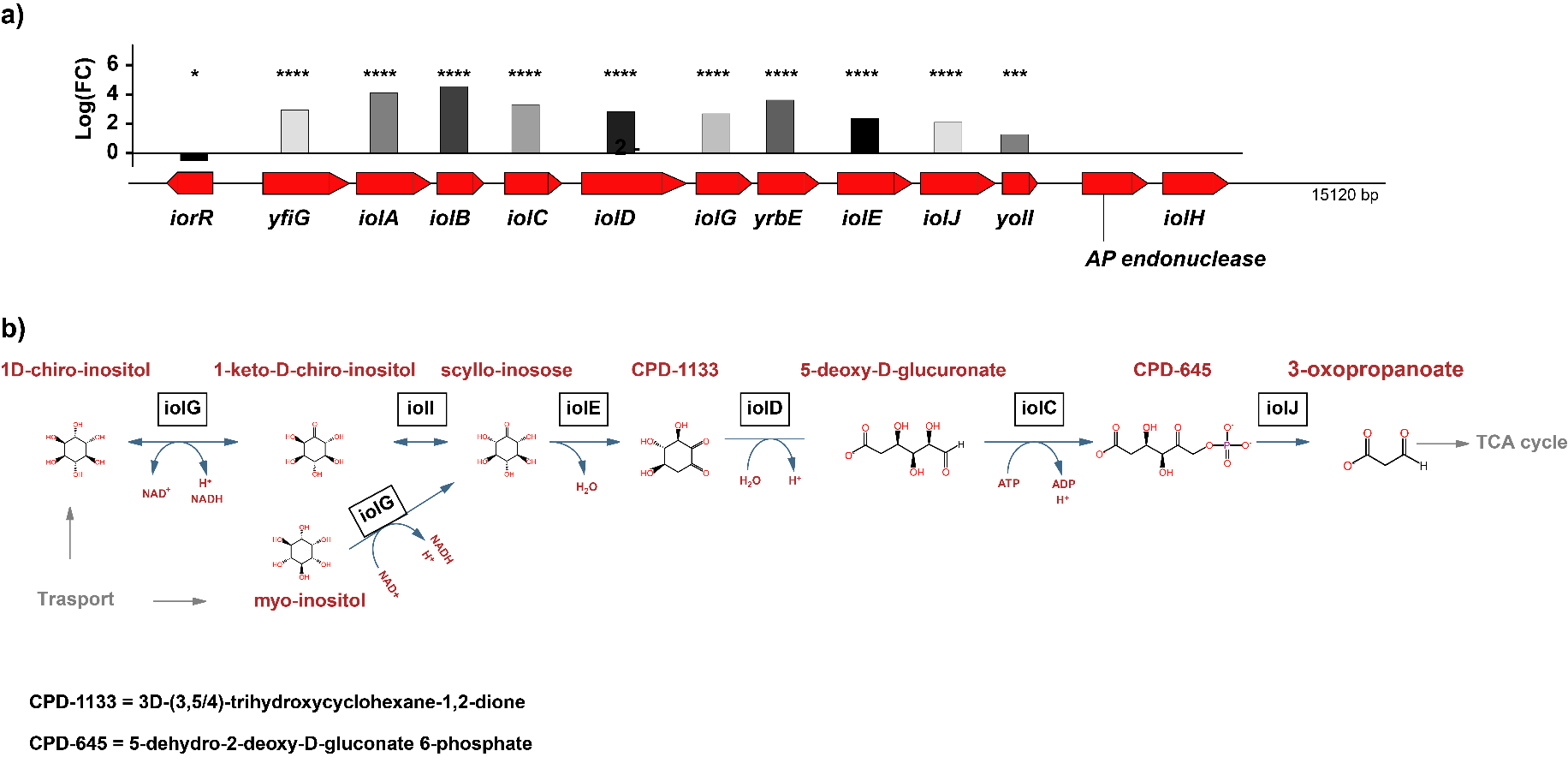


Supplementary figure 4: inositol degradation gene cluster and pathway. a) Inositol degradation gene cluster as present in L. rhamnosus genome. 11 genes of the operon were significantly upregulated in situ relative to in vitro samples (EdgeR, *: FDR adjusted p-value < 0.05, *** FDR adjusted p-value < 0.005, **** FDR adjusted p-value < 0.0001), the bar plot represents log of the expression fold change. Notably, only the transcriptional repressor iolR, predicted to suppress the expression of the operon, was downregulated in situ (adjusted p-value < 0.05). b) Myo- and chiro-inositol degradation pathway, showing the first steps to the degradation of Myo- and chiro-inositol to DHAP and acetyl-CoA. Derived and modified from MetaCyc [90].

Supplementary table 1: Gene Set Enrichment Analysis using the differentially expressed genes (EdgeR, cut-off: FDR adjusted-p-value≤0.05, Log2FC≥2), revealing enrichment of 13 KEGG gene sets, encompassing 11 belonging to the KEGG category “Metabolism” of which 8 belong to “Carbohydrate metabolism”. GSEA, p-value and fold change calculated via FUNAGE-Pro [54]. *: percental of differential expressed genes found in the class.

| KEGG I | KEGG II | KEGG III | p-value | Log_2_(FC) | %* | Genes |
| --- | --- | --- | --- | --- | --- | --- |
| Environmental Information Processing | Membrane transport | ABC transporters | 2.60E-06 | 2.265 | 8% | *rbsA-D, opuAA-AC, oppD, artR* |
| Genetic Information Processing | Translation | Ribosome | 7.60E-12 | 0.980 | 42% | *rpsK, rpsM, rplO, rpmD, rpsE, rplR, rplF, rpsH, rplE, rplX, rplN, rpsQ, rpmC, rplP, rpsC, rplV, rpsS, rplD, rpsT, rpmF and rpmE2* |
| Metabolism | Carbohydrate metabolism | Ascorbate and aldarate metabolism | 9.50E-08 | 3.256 | 63% | PTS ascorbate subunit IIC, *ulaA, sgaT, ulaG,* PTS system Lactose/Cellobiose subunit IIB |
| Metabolism | Carbohydrate metabolism | Fructose and mannose metabolism | 7.60E-11 | 2.339 | 47% | *por, levE, manY, manZI-II, fruK, rhaA, cmtB* |
| Metabolism | Carbohydrate metabolism | Galactose metabolism | 1.10E-08 | 1.807 | 27% | *gatCI-III, dgoD, lacG1, sgcAI-II, kbaY, galK, galT, lacC, lacD2, lacB2, lacA, malL, sgcC, agaC* |
| Metabolism | Carbohydrate metabolism | Glycolysis / Gluconeogenesis | 6.60E-05 | 2.101 | 17% | *fbaAI-III, pgK, bglA* |
| Metabolism | Carbohydrate metabolism | Pentose and glucuronate interconversions | 8.60E-07 | 2.699 | 42% | *uidA, uxaC, uxuA, rhaDI-II, rhaB, ulaE-D* |
| Metabolism | Carbohydrate metabolism | Pentose phosphate pathway | 1.60E-04 | 1.507 | 21% | *kdgK, xyIB, yqeC* and a phosphoketolase |
| Metabolism | Carbohydrate metabolism | Pyruvate metabolism | 1.40E-03 | -0.775 | 20% | *uxaC, rhaD, rhaDII, yuIV, sgbH* |
| Metabolism | Carbohydrate metabolism | Starch and sucrose metabolism | 1.80E-05 | 2.453 | 22% | *glgA, malA, gmuC* and two IIC permease components |
| Metabolism | Lipid metabolism | Fatty acid biosynthesis | 4.50E-06 | -2.559 | 50% | *acpA, fabH,* and two *fabZ* |
| Metabolism | Metabolism of cofactors and vitamins | Thiamine metabolism | 1.80E-05 | 10.726 | 36% | *tenA, thiM, thiE* and a hydroxyethylthiazole kinase |
| Metabolism | Nucleotide metabolism | Pyrimidine metabolism | 8.60E-07 | 1.768 | 27% | *pyrAA, pyrAB, pyrC-F* |
| Protein families: genetic information processing |  | Transcription factors | 3.30E-02 | 1.695 | 6% | *fruR, manR* and a HTH domain protein |
| Protein families: signaling and cellular processes |  | Transporters | 1.00E-06 | 2.261 | 11% | Ammonium transporter, *yojA, frwB, ycel, mntH, crcB, iolF* and *3 unknown* ABC transporters |
| RNA family |  | Non-coding RNAs | 2.50E-02 | 0.190 | 4% | *agaS, cspC, defB, yneF, yulD and a MMPL family protein* |

Supplementary table 2: Gut-Brain Modules present in L. rhamnosus genome. Adjusted p-value and LogFC calculated via edgeR. The contribution enrichment factor is defined as log_2_ of the fraction of all L. rhamnosus reads mapped to a specific category divided by the fraction of all other metatranscriptome reads mapped to the same category. Frequency in reference genomes were extracted from Valles-Colomer et al. [59].

NA: not applicable *: too low reads count

| GBM name | GBM ID | KOs | Genes in L. rhamnosus | FDR adjusted p-value | LogFC | Contribution enrichment factor (GMB) | Frequency in reference genomes of intestinal bacteria [59] |
| --- | --- | --- | --- | --- | --- | --- | --- |
| Tryptophan synthesis | MGB005 | K01696, K01817, K01609, K00766 | LRHA10_v2_0093  LRHA10_v2_0094  LRHA10_v2_0095  LRHA10_v2_0096 | 8.32E-08  1.90E-05  0.0014  0.0026 | 1.988  1.775  1.187  1.173 | -0.593 | 69% |
| 17-beta-Estradiol degradation | MGB031 | K07124 | LRHA10_v2_1357 | 0.0028 | 0.799 | 1.913 | 86% |
| Acetate synthesis I | MGB043 | K00925, K06925, K00656 | LRHA10_v2_0189  LRHA10_v2_0997  LRHA10_v2_1418  LRHA10_v2_2162 | 0.0556  0.1466  0.0002  0.3431 | 0.559  -0.409  1.475  -0.425 | 0.082 | 95% |
| ClpB | MGB029 | K03695 | LRHA10_v2_1359 | 0.0068 | 0.812 | 0.002 | 98% |
| Glutamate synthesis I | MGB006 | K00262 | LRHA10_v2_0599 | 0.3516 | 0.294 | -2.759 | 81% |
| Glutamate synthesis II | MGB007 | K00266 | LRHA10_v2_2511 | 1.13E-14 | 2.966 | -5.582 | 76% |
| Inositol degradation | MGB038 | K00010 | LRHA10_v2_0263 | 4.75E-24 | 3.241 | 5.888 | 10% |
| Quinolinic acid degradation | MGB033 | K01916 | LRHA10_v2_1846 | NA* | NA* | NA* | 97% |
| SAM synthesis | MGB036 | K00789 | LRHA10_v2_0874 | 0.0414 | -0.706 | -0.491 | 98% |
